# Circulating miRNAs as Potential Marker for Pulmonary Hypertension

**DOI:** 10.1371/journal.pone.0064396

**Published:** 2013-05-23

**Authors:** Chuanyu Wei, Heather Henderson, Christopher Spradley, Li Li, Il-Kwon Kim, Sandeep Kumar, Nayeon Hong, Alejandro C. Arroliga, Sudhiranjan Gupta

**Affiliations:** 1 Division of Molecular Cardiology, Department of Medicine, College of Medicine, Texas A&M Health Science Center, Scott & White, Central Texas Veterans Health Care System, Temple, Texas, United States of America; 2 Scott & White Memorial Hospital, Temple, Texas, United States of America; Wageningen UR Livestock Research, The Netherlands

## Abstract

MircoRNAs (miRNAs) are small non-coding RNAs that govern the gene expression and, play significant role in the pathogenesis of heart failure. The detection of miRNAs in circulation of pulmonary hypertensive (PH) human subjects remains elusive. In the current study, we determined the pattern of miRNAs of mild-to-severe human PH subjects and, compared them with the control subjects by miRNA array. Blood was obtained using fluoroscopic and waveform guided catheterization from the distal (pulmonary artery) port of the catheter. A total 40 human subjects were included in the study and, the degree of PH was determined by mean pulmonary arterial pressure. Among several miRNAs in the array, we validated 14 miRNAs and, the data were consistent with the array profile. We identified several novel downregulated miRNAs (miR-451, miR-1246) and upregulated miRNAs (miR-23b, miR-130a and miR-191) in the circulation of PH subjects. Our study showed novel set of miRNAs which are dysregulated in PH and, are directly proportional to the degree of PH. These miRNAs may be considered as potential biomarker for early detection of PH.

## Introduction

MicroRNAs (miRNAs) are small, endogenously expressed noncoding RNAs that regulate gene expression at post-transcriptional level, via degradation or translational inhibition of their target mRNAs [1], [2]. MiRNAs are ≈22 nucleotides in length which bind to the 3′ untranslated region of specific target genes and thereby suppress/inhibit the translation of target genes. Functionally, an individual miRNA can regulate the expression of multiple target genes [3], [4]. Although, the exact mechanism is unclear, it is reasonable to accept a “seed region” of 3 to 8 nucleotide of miRNA might be accountable for the interaction [3].

Pulmonary hypertension (PH) is a progressive pulmonary vascular disease with high morbidity and mortality. PH results in right ventricular hypertrophy (RVH), progressive fibrosis and RV failure; and low cardiac output leading to increase morbidity, and mortality [5], [6]. In the clinical setting, the disease is often detected in later stages marked by full-blown RV failure and, the treatment regimen is limited that includes the use of prostaglandin analogues, endothelin receptor antagonist and PDE5 which works primarily in the vasculature as a vasodilator and anti-proliferative [7], [8], [9]. The pathological manifestation of PH is remodeling of pulmonary arteries which increased proliferation of pulmonary artery smooth muscle cells and dysfunction of pulmonary artery endothelial cells resulting gross vascular remodeling. There is accumulating evidence for the critical role of miRNA in diverse cardiovascular remodeling that include development, vessel wall homeostasis, angiogenesis and vascular injury [10], [11], but, the miRNA profiling in vascular diseases are limited.

Recent studies have shown that miRNAs are circulating freely in the mammalian blood and can be predicted as “biomarker” for early diagnosis of acute myocardial infarction and heart failure in humans [12], [13], [14], [15], [16], [17]. The molecular mechanism of presence of stable miRNA in the circulation is currently elusive. However, several evidences suggest that miRNAs are secreted as micro vesicles or exosome and apoptotic bodies that may be responsible for release the miRNAs into the circulation [18], [19], [20], [21] and, are extremely stable in the blood or serum [22]. Although, recent studies identified circulatory miRNA in coronary artery disease [23], [24], but, the signatory profile of miRNAs is not established in human PH subjects. Identification of circulatory miRNAs in PH subjects may provide a novel therapeutic intervention opportunity. The objective of this study is to identify the miRNA(s) signature in circulation of human PH subjects. This study is the first to identify novel miRNAs in human PH which, could act as potential biomarker for the diagnosis of PH.

## Methods

### Patient characteristics

The patients were visited at Scott & White Hospital, Temple, TX with PH diagnosed by right heart cath. The consent was obtained from all participants before enrolled in this study. Forty consecutive patients undergoing right heart catheterization for diagnosis of pulmonary hypertension or monitoring of disease progression and response to therapy were enrolled in the study. The study protocol was approved by the ethics committee of Scott & White Hospital that conforms to the principles outlined in the Declaration of Helsinki. All clinical samples were obtained with approval of the Scott & White Hospital research ethics committees. The written consents were obtained from each patient for this study and were kept on file. The consent procedure was approved by S & W Ethics' committee. The protocol (#100029) was approved by the Scott & White Healthcare Institutional Review Board.

After fluoroscopic and waveform guided catheterization of the pulmonary artery, approximately 20 ml of blood was obtained from the distal (pulmonary artery) port of the catheter with the balloon deflated. The blood was then injected into purple top tubes and transported to the laboratory for processing as described below.

**Figure 1:**
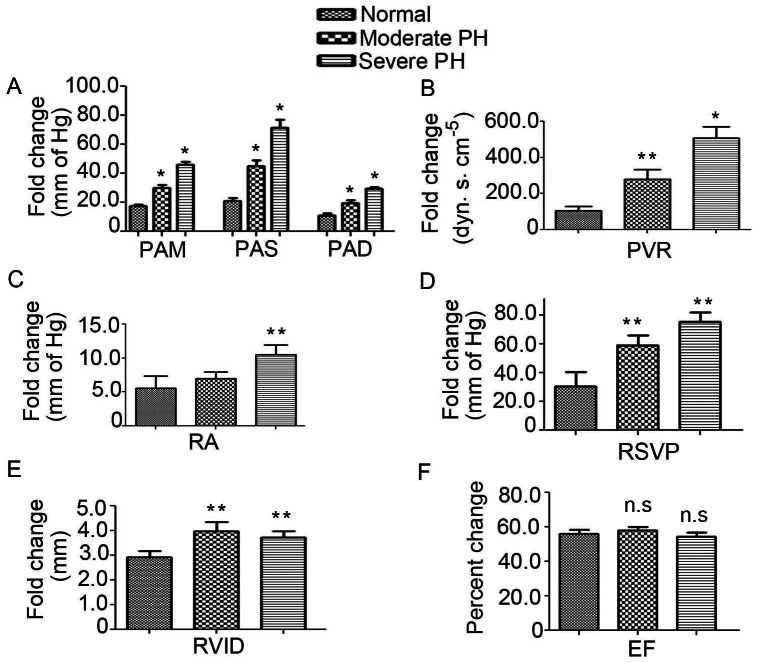
Characterization of human PH patients profiles. **A.** The PAM, PAS and PAD were determined by catheterization as described in material and methods. **B.** Determination of profiles for pulmonary vascular resistance (PVR), **C.** Right atrial pressure (RA), **D.** Right ventricular systolic pressure (RVSP), **E.** Right ventricular internal diameter (RVID) and **F.** Ejection fraction (EF). Data represent means ± SEM. N = 8 for control, 15 for moderate PH and 17 for severe PH group. *denotes p<0.001 and ** denotes p<0.05 compared to the healthy subjects, n.s. is non-significant.

Patient characteristics are summarized in table 1. The patients were stratified as having mild, moderate, or severe PH on the basis of mean pulmonary artery pressure. Using a cutoff of 40 mmHg and above for severe disease, 35 mmHg and above for moderate disease and above 25 mmHg for mild disease, 16 patients were identified as severe, 15 patients were considered as moderate and, 1 was excluded due to right heart failure. In this group, disease severity correlated with PVR, RAP, RVSP, and RVID (Figure 1). The remaining 8 were defined as controls on the basis of normal resting hemodynamics. Additionally, we have included 8 more healthy subjects in control group for validation of selected miRNA analysis. This group of patients had undergone catheterization to rule out PH as a cause of dyspnea. The classification was based with pulmonary hypertension World Health Organization (WHO) clinical classification system (Dana Point 2008). The PH has been defined as an increase in mean pulmonary arterial pressure (PAP) >25 mmHg at rest as assessed by right heart catheterization (RHC) as per the guidelines for diagnosis and treatment of pulmonary hypertension as described [25]. This value has been used for selecting patients in this study. It is of note that our sample population is a heterogeneous in nature. But, in our patient group, we observed that all had elevated mean PAP and were associated with the symptoms of pulmonary hypertension as per the guidelines of WHO clinical classification. Of the patients with pulmonary hypertension, 11 were diagnosed with revised (Dana Point) group 1 disease or pulmonary arterial hypertension. 7 were diagnosed with group 2 PH showing to left sided cardiac dysfunction, 8 were diagnosed with group 3 PH owing to structural lung disease, and 1 was diagnosed with group 4 PH secondary to chronic thromboembolic disease. A detailed diagnostic parameters and associated condition of PH subjects are shown in Supporting Information S1.

**Table 1:** The one-way ANOVA was performed to determine the significance level among the groups.

	Control	Moderate PH	Severe PH	P value
PAM (mm of Hg)	17.57±1.17	29.9±1.94	46±2.2	p<0.0001
PAS (mm of Hg)	21.0±2.19	45.2±3.67	71.67±5.5	p<0.0001
PAD (mm of Hg):	11.0±1.39	19.4±2.1	29.58±0.85	p<0.0001
RA (mm of Hg)	5.57±1.77	7.0±0.96	10.5±1.44	p<0.05
PVR (dyn*.s*.cm^−5^)	105.6±24.64	280.3±53.74	508.4±62.73	p<0.0001
RVSP (mm of Hg)	30.33±10.17	59.29±6.83	75.6±6.83	p<0.05
RVID (cm)	2.92±0.24	3.98±0.35	3.73±0.24	p<0.05
EF (%)	56.14±2.20	57.92±1.99	54.5±2.5	p = n.s

P<0.0001 was observed for PAM, PAS and PAD, p = 0.058 for RA, p = 0.0002 for RVR, p = 0.0007 for RVSP, p = 0.088 for RVID and p = n.s for EF.

Of the patients included in group 1, 4 were treatment naïve, 3 were taking calcium channel blockers alone, 2 were taking sildenifil and ambrisentan, 1 was taking bosentan and calcium channel blockers, and 1 was taking tadalafil and calcium channel blockers.

The miRNA array was performed using the PH subject whose mean PAP was 53 mm Hg and diagnosed as idiopathic pulmonary hypertensive patient. The samples we validated in our cohort study were considered as pulmonary hypertensive. However, these subjects were also exhibited symptoms associated with PH like COPD, interstitial lung disease, obstructive lung disease, scleroderma, etc. There was no morbidity occurs so far in this study group; therefore, we believe that the changes of miRNA in the blood were associated with PH.

During the right heart catheterization, approximately twenty milliliters of blood were obtained from the pulmonary artery, processed and, the buffy coats were prepared and were stored at −80°C.

### MiRNA isolation

The miRNA was isolated using the PH subject whose mean PAP was 53 mmHg and diagnosed as idiopathic pulmonary hypertensive patient. The control subjects miRNA was isolated from the subjects whose mean PAP was 20 mmHg. Isolation of miRNA was done using miRNeasy Mini Kit (Qiagen, California, USA) as per manufacturer's instructions. Briefly, Twenty milliliters of blood were collected from study subjects in PAXgene Blood RNA tubes (BD, USA). The buffy coats (250 µL each) were homogenized in 700 µL of QIAzol lysis reagent. After addition of 140 µL chloroform, the homogenate was separated into aqueous and organic phases by centrifugation. The upper aqueous phase (approximately 320 µL) containing the RNA was extracted, and 100% ethanol (approximately 525 µL) was added to provide appropriate binding conditions for all RNA molecules from 18 nucleotides (nt) upwards into the miRNeasy columns. This was then applied to the RNeasy Mini spin column, where the total RNA was bound to the membrane and phenol and other contaminants were washed away by washing with the wash buffer (700 µl Buffer RWT) and subsequently, twice with 500 µl of Buffer RPE. Total microRNA was then eluted in 20 µL RNase-free water.

### Microarray processing and Analysis

RNA was quantified and assessed using the RNA 6000 Nano Kit and 2100 Bio analyzer (Agilent Technologies UK Ltd, West Lothian, UK). MiRNA array was performed and analyzed by LC Bioscience, Austin, TX, USA. In brief, this analysis was performed on the Agilent miRNA array, using the labeling kit, reagents and methods recommended by the manufacturer. Samples for miRNA profiling studies were processed by LC Bioscience (Austin, TX), according to standard operating procedures in the GLP-compliant services laboratory. Total RNA is first dephosphorylated and then the pCp-Cy3 labeling molecule is ligated to the 3′ ends of the RNA molecules. The labeled RNA is purified using BioSpin6 (Bio-Rad, Hercules CA). Hybridization, washing, staining, imaging, and signal extraction were performed according to Agilent-recommended procedures. The data represent as log_2_ value. Paired t-test or ANOVA were performed to find statistically significant difference between the groups and p<0.05 was considered as a significant.

Average values of the replicate spots of each miRNA on the microarray were normalized using global normalization. The correction factor was calculated by dividing the sum of intensities of each sample by the average of all the samples. The normalized values were calculated by multiplying average intensities of each miRNA with the correction factor. The JMP 6.0.0 software (SAS Institute Inc., Cary, NC) was used for hierarchical clustering, generation of heat maps, and principal component analysis (PCA). The miRNA array data is MIAME compliant has been submitted to the Gene expression Omnibus server at NCBI vide GEO no GSE4415.

### Complementary 1^st^ strand-DNA synthesis and quantitative real-time PCR

The samples we validated in our cohort study were considered as pulmonary hypertensive. However, these subjects were also exhibited symptoms associated with PH like COPD, interstitial lung disease, obstructive lung disease, scleroderma, etc. There was no morbidity occur so far in this study group, therefore, we believe that the changes of miRNA in the blood were associated with PH.

To confirm the miRNA array data, we performed a qRT-PCR using buffy coat. A total of 40 samples were studied as a cohort basis. Two hundred nano-grams of each subjects RNA enriched for small species with length <200 bases were reverse-transcribed into first strand cDNA using an RT^2^ miRNA First Strand Kit (SA Biosciences, Frederick, MD). The cDNA was mixed with RT^2^ SYBR Green/ROX qPCR Master Mix and the mixture was added to individual specific primers and, has- miR-U6 (Housekeeping micro-RNA) as per manufacturer's instruction in technical duplicates. PCR was performed on a Stratagene MX 3005 Real-Time PCR System (Stratagene, CA). The following thermal cycle was used 95°C, 10 min, 40 cycles of (95°C, 15 sec; 60°C, 40 sec; 72°C, 30 sec). ROX was used as internal reference dye.

### Data Analysis

Threshold cycle (Ct) for each miRNA was extracted using MXPro software (Stratagene) by setting threshold and automatically defined baseline. The raw *C*_t_ was normalized using the quintile method to remove the variations from the housekeeping genes. The fold change for each miRNA was calculated using ΔΔ*C*_t_ method. The data for the expression of these miRNAs was normalized to 10^6^ copies of housekeeping has-miR-U6. If the expression of miRNAs of interest showed a copy number of ≤1 per 10^4^ copies of has-miR-U6, it was termed as “not detected.”

### Statistical Analysis

Statistical analyses were performed using GraphPad Prism 5.0 statistical package for Windows (GraphPad Software Inc., La Jolla, CA). Data are presented as mean ± SEM unless indicated otherwise and, plasma miRNA levels are presented as fold-change relative to the healthy subjects. Linear regression analyses were used to correlate quantitative variables after normalization of non-Gaussian variables by log transformation. Comparisons between 2 groups were performed using Student t tests for Gaussian data or Mann-Whitney tests for non-Gaussian data. The P-value (P<0.05) was considered as statistically significant differences between the two groups. Results were also analyzed by one-way ANOVA for comparative analysis in between the groups (moderate and severe PH human subjects) with those of healthy control subjects.

## Results

### Patient characterization and evaluation

We have included mean pulmonary artery pressure (mPAP), right atrial pressure (RA), pulmonary vascular resistance (PVR), pulmonary artery systolic pressure (PAS) and pulmonary artery diastolic pressure (PAD) for moderate-to-severe PH in human subjects. Our data showed that mPAP, PAS and PAD were significantly increased in the PH patients compared to the controls (Fig. 1A). Additionally, we observed that the RA, pulmonary vascular resistance (PVR), right ventricular systolic pressure (RVSP) and right ventricular internal diameter (RVID) were significantly increased compared to the control subjects (Fig. 1B). It is of note that above functional parameters were increased as the disease progressed or the severity (Fig. 1). The detail analysis of the above parameters are shown below in the table 1

### PH induces changes in miRNA profile in circulation

**Figure 2:**
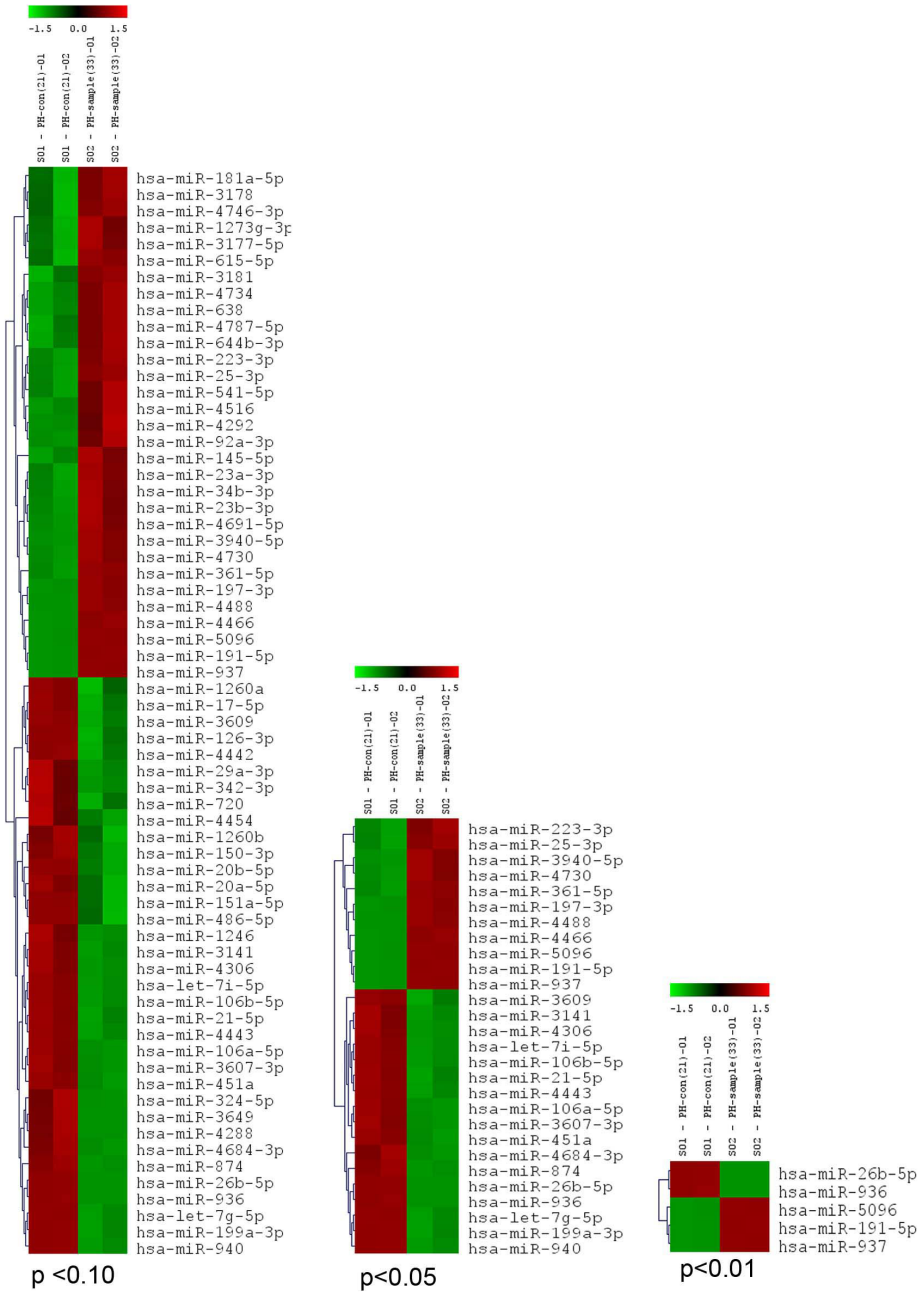
Heat map showing the expression of dysregulated miRNAs in control and PH human subjects. MiRNA microarray data was prepared using data from the miRNA microarray performed using samples from PH human patients and compared with healthy controls. The signal difference (log_2_) is plotted versus the level of statistical significance (−log_10_ p-value). Color and spot diameter depicted information about mean expression level and percent detected, respectively, in static plots.

This is the first report where we observed differential miRNA expression in patients with PH and compared with healthy individuals who did not show the mean arterial pressure greater than 25 mm of Hg by miRNA microarray. We observed that several miRNAs were differentially expressed in the PH patients compared to control subjects (Figure 2). The data analysis package from LC Sciences was used to prepare data-rich volcano plots between, which show p-value and fold-change between two groups for all miRNAs. Color and spot diameter depicted information about mean expression level and percent detected, respectively in the heat maps (Figure 2). The arrays were based on Sanger miR Base release 18.0 databases. For two-color experiments, the ratio of the two sets of detected signals (log_2_- transformed, balanced) and the p values of the t-test were calculated.

We validated a set of miRNAs in our cohort study. We choose the following miRNAs for validation:

MiR-21, miR-23a, miR-26a, miR-29, miR-34b, miR-191, miR-451 and miR-1246 were derived from the miRNA array analysis (Figure 2). We also have included few cardiac specific miRNAs (e.g. miR-1, 133b and 208b) as to test whether they are circulating in PH subjects. Furthermore, we included another miRNA which was reported to be involved in PH (e.g. miR-204). Finally, we tested two other miRNAs which had target genes that were associated with PH. These miRNAs are miR-30b which has target for Notch1 and Bcl_2_ and, miR-130a has target for BMPR1b and SMAD2. It is of note that these miRNAs were not detected in the miRNA array possibly because of low level of detection limit.

### MiR-1, miR-26a, miR-29c, miR-34b, miR-451 and miR-1246 are downregulated in PH subjects

**Figure 3:**
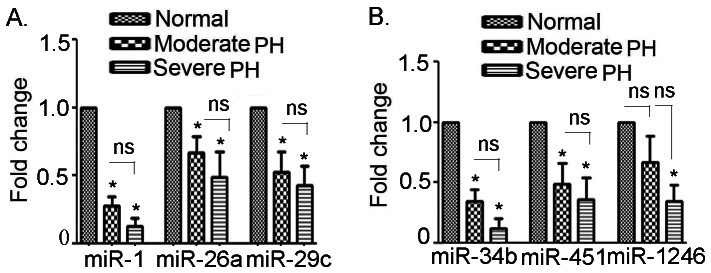
Real time-PCR based validation of downregulated miRNAs in the buffy coat of healthy human subjects vs. PH patients. **A.** Expression of miR-1, miR-26a and miR-29c **B.** Expression of miR-34b, miR-451 and miR-1246. Data represent means ± SEM. N = 16 for control, 15 for moderate PH and 17 for severe PH group. *denotes p<0.05 and ** denotes p<0.01 compared to the healthy subjects.

We determined the expression of circulatory miRNAs in control, moderate PH and severe PH subjects. The expressions of miR-26a, miR-29c, miR-451 and miR-1246 were declined to 0.66±0.12, 0.52±0.14, 0.49±0.16 (p<0.05) and 0.67±0.21-folds (p = ns) respectively, in moderate PH subjects, compared to the control subjects. The expressions of the above miRNA were further declined to 0.49±0.18, 0.42±0.14, 0.34±0.14 and 0.36±0.17-fold respectively (p<0.05), in severe PH subjects, compared to the control subjects (Fig. 3A and 3B). The expression of miR-34b and miR-1 showed strongest declined pattern among the PH subjects. The expression of miR-34b was 0.34±0.1 and 0.12±0.08 (p<0.05) in moderate and severe PH respectively, compared to the control counterparts. The expression of miR-1 showed 0.27±0.06-fold in moderate PH and elicited a sharp decline to 0.13±0.05-fold (p<0.05) in severe PH subjects, compared to the control subjects (Fig. 3 B). The expression of the above miRNAs was not significant when compared the values between moderate *vs.* severe PH.

### Mi-130a, mir-133b, miR-191, miR-204 and miR-208b are highly elevated in PH subjects

**Figure 4:**
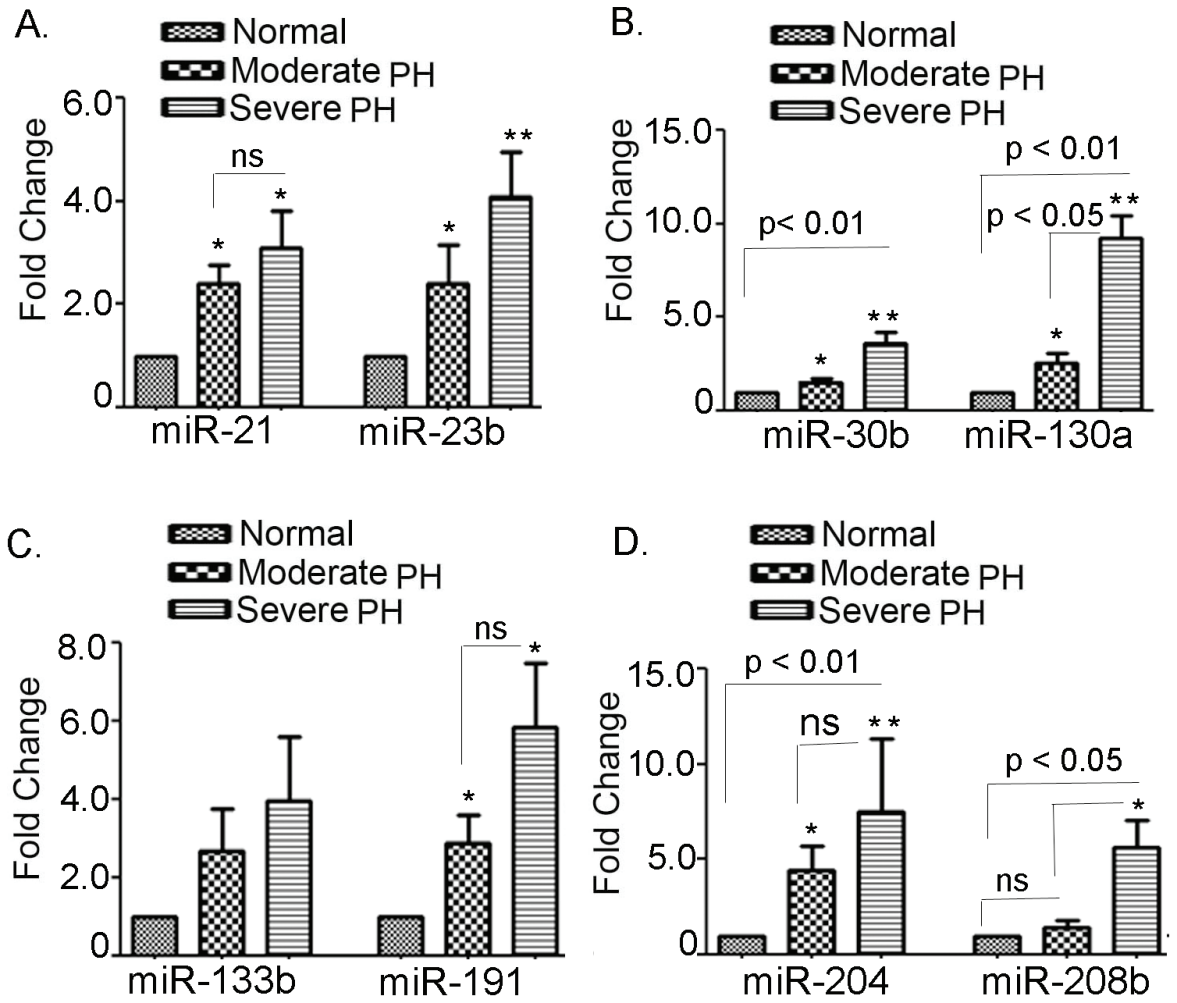
Real time-PCR based validation of upregulated miRNAs in the buffy coat of healthy human subjects *vs.* PH patients. **A.** Expression of miR-21 and miR-23a, **B.** Expression of miR-30b and miR-130a, **C.** Expression of miR-133b and miR-191, **D.** Expression of miR-204 and miR-208b. Data represent means ± SEM. N = 16 for control, 15 for moderate PH and 17 for severe PH group. *denotes p<0.05 and ** denotes p<0.01 compared to the healthy subjects.

The expression of miR-130a, miR-133b and miR-191 were increased significantly in all PH subjects and, showed further pronounced in severe PH subjects. The expression of miR-130a showed a 2.54±0.56-fold (p<0.05) enhancement in moderate PH where severe PH showed a sharp increment of 9.27±1.19-fold (p<0.01), respectively. In moderate PH subjects, the expression of miR-133b was 2.70±1.07-fold and 3.96±1.63-fold (p = ns) in moderate and severe PH subjects, compared to the control subjects. Likewise, in the moderate PH group, the expression of miR-191 was 2.88±0.71-fold and was further increased to 5.83±1.66-fold (p<0.05) in severe PH subjects, compared to the controls (Fig. 4A). The expression of miR-204 was increased to 4.42±1.29-fold (p<0.05) in the moderate PH subjects and was further elevated to 7.48±3.8 fold (p<0.01) in severe PH subjects, compared to their controls (Fig. 4B). The expression of miR-208b was 1.44±0.35 (p = ns) in moderate PH subjects and was 5.62±1.46 (p<0.05) in severe PH subjects, compared to their controls (Fig. 4B). It is of note that the expression of miR-133b, miR-191 and miR-204 were not significant in severe PH subjects, compared to their moderate counterparts. However, the expression of miR-130a and miR-208b were significant in severe PH subjects, compared to their moderate counterparts.

### MiR-21, miR-23b and miR-30b are moderately elevated in PH subjects

The expression of miR-21 and miR-23b were moderately increased in all PH subjects but showed more pronounced in severe PH subjects. In moderate PH subjects, the expression of miR-21 was 2.4±0.36-fold and 3.09±0.71-fold (p<0.05) in severe PH subjects, compared to the control subjects. Likewise, in the moderate PH group, the expression of miR-23b was 2.39±0.76-fold (p<0.05) and 4.07±0.88-fold (p<0.01) increased in severe PH subjects, compared to the controls (Fig. 4B). The expression of miR-30b was 1.54±0.22-fold (p<0.05) and 3.58±0.63-fold -folds (p<0.01) in moderate and severe PH, respectively (Fig. 4 B). It is of note that the expression of miR-23b and miR-30b but, not the miR-21, were significant in severe PH subjects; compared to their moderate counterparts.

### Influence of gender in miRNA expression in PH subjects

**Figure 5:**
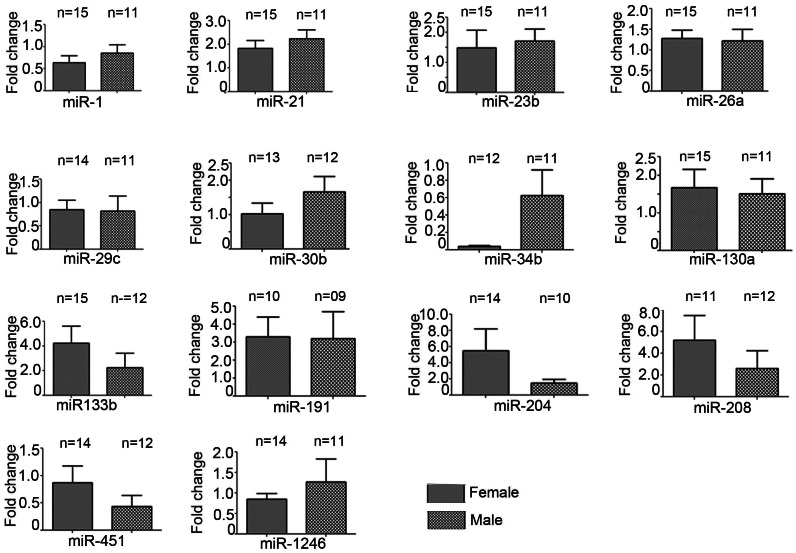
Effect of gender on dysregulated miRNAs miRNAs were detected in the validation cohort of PH by RT-PCR and was normalized using has-U6 housekeeping gene. Data represent means ± SEM, n = 12–15 for female PH subjects and n = 10–12 for male PH subjects.

In order to determine whether gender has any influence in circulatory miRNA expression, we determined all validated miRNAs as gender based cohort study. We compared the miRNA expressions pattern in male and female PH subjects separately; and both the genders (Fig. 5). Among the downregulated miRNAs; miR-1, miR-29c, and miR-34b showed more declined expression in the female subjects, compared to the male counterpart. In contrast miR-451 and miR-1246 showed sharp declined expression in the male subjects (Fig. 5). The miR-26a did not show any significant pattern of changes between male and female subjects. Likewise, among upregulated miRNAs, miR-23b and miR-30b showed more incremental expression in the male subjects, compared to the female subjects. In contrast, miR-21, miR-133b, miR-191and miR-208b showed enhanced expression in the female subjects, compared to the male counterpart (Fig. 5). The expression of miR-130a and miR-204 did not show any significant pattern changes among the male and female subjects, compared to their total expression. The severity of PH did not show any significant influence between the male and female subjects.

## Discussion

The present study for the first time demonstrates that PH induces novel circulatory miRNA derived from human PH subject. Recent evidences indicate that freely circulating miRNAs may be informative in the diagnostic setting and have the potential to be used as biomarkers. Our study demonstrates that in human subjects with diverse clinical settings in PH are associated with distinct dysregulation of miRNAs in the circulation. It is of note that PH is associated with other diseases like COPD, scleroderma, obstructive lung diseases, *etc*, and, may therefore, consider as an idiopathic type. However, our data are consistent with respect to mPAP elevation and severity of PH which is the hallmark for PH. The observation may provide early detection of PH in human serum.

Our study reveals that in human subjects with moderate to severe PH are associated with significant downregulation of plasma levels of circulatory miR-1, miR-26a and miR-29c. The kinetics of the changes differed from moderate to severe PH human subjects as we observed further downregulation of the above miRNAs in severe PH. Targetscan analysis showed that miR-26a and miR-29c had target genes for collagens which involved in pro-fibrotic events and fibrosis. It is of note that the patient population include in this study suffered from lung fibrosis, interstitial lung disease and, RVH that lead to cardiac fibrosis. The miR-1 has target genes for Notch3 which is upregulated in PH in human as well as in rat model may indicate a functional correlation [26]. Likewise, miR-29 family directly target at least 16 extracellular matrix genes, providing a strong evidence for anti-fibrotic effects in related organs including heart [27]. TargetScan and PicTar analysis showed that miR-26a showed target for collagen type I and CTGF gene and miR-451 showed target for titin which are markers for pro-fibrotic process. The miR-1246 showed target for caveolin1, GSK3ß and cadherin 2 genes. Together, our data indicate a possibility of functional correlation between the circulatory miRNA and distant organ dysfunction. The reduction of these miRNAs in circulation are surprising as we expect that either endothelial or cardiac cell activation, the miRNAs should be shedding off as microparticle or apoptotic bodies, thereby, elevating the level in circulation [18], [19], [20], [21]. A recent analysis indicated that miRNAs can be transferred from cell-to-cell by lipid-based carriers and affect gene expression or can be delivered by apoptotic bodies [21], [28]. Therefore, we can speculate the attenuation of these miRNAs might be taken up by the damaged vessels and elicit less abundant in the circulation.

Our data indicated a set of upregulated miRNA in the circulation of PH subjected compared, to the controls. During moderate PH, miR-21, miR-23b, miR-130a, miR-491 and miR-1246 are moderately upregulated but, are more pronounced in severe PH, compared to the controls. Our data also revealed another set of miRNAs that include miR-133b, miR-204 and miR-208b which were significantly upregulated in all moderate PH subjects but, showed further increment in severe PH subjects. Among them, miR-208b and miR-133b are considered to be cardiac specific suggesting that they may be released from the injured non-cardiac muscles [29], [30], [31], [32]. The upregulation of miRNA appears to have some functional relevance when analyzed through TargetScan, MiroCosm or PicTar databases. Interestingly, miR-23b and miR-130a showed target genes for BMPR1b which is degraded during PH and considered as a predictor for PH [33], [34]. Our recent data support this observation where we showed downregulation of BMPR1b, Id1, Id3 and Smad2 in monocrotaline induced mouse PH model [35], but, the functional correlation between the circulatory miRNA and tissue level expression has not been determined. Likewise, the potential role of miR-21 has been defined in few recent studies in PH and vascular remodeling [36], [37], [38]. The expression of miR-21 has been demonstrated to be downregulated in mouse model or in human subjects. Moreover, in a recent study by Courboulin A *et al* showed the downregulation of miR-204 in human PH subjects and in experimentally induced PH rat model and discovered a new regulatory pathway involving miR-204, which was critical to the etiology of PH. The study showed that delivery of synthetic miR-204 to the lungs of animals with PH significantly mitigated disease severity [39]. Our data corroborated with the finding of upregulation of both miR-21 and miR-204. The upregulation of these miRNAs can be explained by the release from injured vessels into circulation. It may be speculated that miRNAs may leak into the blood stream during injury and are steadily present as an elevated level in the circulation. The biological correlation between circulatory miRNA and the distant organ expression has not been established and warrants further investigation. However, it was reported that after drug-induced liver injury, miRNAs elevated in plasma were consistently downregulated in corresponding liver suggesting the intriguing possibility of a cellular survival mechanism in which, during stress, undesirable miRNAs are actively excreted [15]. A recent study further suggests that miRNA can be produced by one cell type in a different organ and, can be transported like hormones to the effector site for action [40]. Therefore, it is reasonable to speculate that these miRNAs are released either form the RV or the damaged lungs or vessels due to elevated PH. Interestingly, we observed a variation of few dysregulated miRNAs in the male and female subjects which opens a new avenue whether gender has any influence in the degree of expression in the circulation.

Together, these data indicated a potential use of these miRNAs as an early detection biomarker of PH. From this study, we present evidence that a set of increased plasma miRNA level that include miR-21, miR-130a, miR-133b, miR-191, miR-204 and miR-208b, can be used as biomarker for assessment of PH. On the contrary, our data revealed a set of declined miRNAs which includes miR-26a, miR-29c, miR-34b and miR-451and; can be used as biomarker as well. These findings are of vital importance for the diagnostic applicability of plasma miRNAs in clinical settings and in particular, where altered distributions of biomarkers, combined with co-morbidities such RVH, pulmonary fibrosis, are common. Alternatively, the significant decrease of miR-1, miR-26a and miR-29c level in PH has potential diagnostic significance.

In summary, the current study provides for the first time the detection of circulatory miRNAs in PH human subjects. We report that initial PH induced damage of endothelial cells marked by increased mPAP and RA initiates dysregulation of miRNAs in the organ or tissue architecture which then shedding off and, come into the circulation. Our study suggests that a specific set of dysregulation (up- and down regulation) of miRNAs originating from either cardiac or vascular bed can be detected in the circulation. Our study includes a diverse PH cohort, but, it is noted that all subjects are diagnosed with PH. We, therefore, assume that changes of miRNA in circulation are primarily due to elevated PH. The dysregulation of these miRNAs may serve as therapeutic target for drug invention or early diagnostic tool for PH, PH induced RVH and interstitial lung disease. Further experimental studies are required to define the synergistic association between the tissue expressions versus circulation in patient population. Although circulatory miRNA offers a great potential for the diagnosis; however, precise quantification, normalization and larger cohort studies are warranted. These parameters are necessary for accurately translating the findings into clinical biomarkers.

### Limitation of the study

Our study provides the first evidence of circulating miRNAs in the setting PH may use as an early detection for PH. Nevertheless, the data presented here are based on limited study population and, the results need to be confirmed in a larger cohort study. Furthermore, the dysregulation miRNA in PH remains to be tested whether these circulatory miRNAs have any influence in the target genes. Elucidating the possible biological role of PH specific miRNAs in the circulation is beyond the scope of this study. However, considering the recent reports describing cell-to-cell transport of miRNAs [41] and miRNAs as paracrine signaling molecules [21], one might speculate that the presence of miRNAs in circulation is not merely a by-product of tissue damage, but also implies a functional role for these molecules.

## Supporting Information

Supporting Information S1 Patients Profile.
(PDF)
