# Supplementary material for: Circulating miRNAs as Potential Marker for Pulmonary Hypertension
Source: PLoS One. 2013 May 23;8(5):e64396. doi: 10.1371/journal.pone.0064396 (PMC3662705; doi:10.1371/journal.pone.0064396)
Supplement: Supporting Information S1 — Patients Profile. (PDF) [file pone.0064396.s001.pdf]

Age	Sex	ID	FC	Associated Condition	RA	PAS	PAD	PAM	PCW	SAP	COTd	CITd	COF	CIF	RVR	VDC	O2	DPC	
71	Female	40	3	Scleroderma	3	113	34	67	4	119	5.8	3.67	3.62	2.29	868.0	Not Done	92.00	1.4.1	
45	Male	23	2	Idiopathic PAH	12	95	33	53	14	129	6	2.73	4.48	2.04	519.0	Negative	100.00	1.1	
74	Female	1	4	Idiopathic PAH	18	73	32	48	19	127	2.9	1.83	2.88	1.82	799.0	Not Done	95.00	1.0	
51	Female	7	3	PAH	11	77	32	48	9	93	4.6	2.47	3.73	2.00	678.0	Negative	95.00	4.0	
76	Female	22	3	Interstitial Lung Disease	10	68	32	45	28	157	3.2	1.88	2.62	1.54	424.0	Not Done	96.00	2	
51	Male	17	3	None	19	77	29	44	7	133	4.9	2.18	4.84	2.16	603.0	Negative	91.00	1.0	
51	Male	28	3	Portal Hypertension	7	62	29	42	15	125	6.7	2.96	5.66	2.50	322.0	Not Done	96.00	1.43	
63	Female	34	3	Obstructive Sleep Apnea	11	69	27	42	13	137	5	2.14	3.31	1.41	463.0	Negative	95.00	3.2	
71	Male	38	3	Lymphoid Vasculitis	3	68	26	42	11	148	8.3	3.71	9.26	4.14	288.0	Negative	94.50	1.1	
61	Female	3	3	Pulmonary Hypertension	13	59	29	41	18	121	4.9	2.41	4.75	2.34	375.0	Not Done	93.00	2	
67	Female	13		Diastolic Heart Failure	12	33	27	40	28	144	7.3	3.58	4.61	2.26	131.0	Not Done	95.00	3.2	
82	Female	27		Idiopathic PAH	7	66	25	40	10	111	3.8	2.38	2.67	1.67	631.0	Negative	95.00	1.1	
51	Male	33		Obstructive Lung Disease	5	59	30	39	7	134	6.1	3.51	3.57	2.06	419.0	Positive	91.00	3.1	
55	Female	25		OSAS; Morbid Obesity	11	58	22	38	22	140	10	3.90	6.52	2.60	120.0	Not Done	94.00	2	
77	Female	37		Interstitial Lung Disease, COPD	5	63	26	38	6	162	4.3	2.70	2.11	1.33	595.0	Positive	99.00	3.3	
63	Female	2		Idiopathic PAH	8	58	21	36	10	131	5.9	3.37	5.91	3.37	352.0	Positive	96.00	1.0	
72	Female	12		LV Dysfunction/Increased Output	12	48	25	35	19	160	10.3	4.72	8.19	3.75	124.0	Not Done	95.00	2.2	
67	Male	18		COPD/ILD	1	55	24	34	1	108	6.2	2.92	6.09	2.87	425.0	Negative	90.00	3.0	
71	Male	32		Interstitial Lung Disease	10	57	22	34	7	127	3.9	2.34	2.18	1.31	553.0	Negative	100.00	3.2	
66	Female	36		Diastolic Dysfunction, OSAS	10	49	27	34	18	141	6.2	2.92	3.00	1.41	206.5	Not Done	95.00	2.2	
64	Female	30		Scleroderma	6	42	21	28	12	150	9.5	4.41	5.76	2.67	222.0	Negative	97.00	1.4	
56	Female	19		PAH; HOCM	17	45	11	26	24	118	6.2	4.39	2.77	1.96	25.8	Not Done	99.00	2.0	
78	Male	31		None	4	50	15	26	11	134	5	2.25	4.46	2.00	239.0	Partial	97.00	1.1	
62	Female	10		Scleroderma/ILD	2	36	16	25	7	159	5.2	2.83	4.41	2.40	276.0	Not Done	96.00	3.2	
58	Female	15		Structural Lung Disease	6	29	20	25	12	115	5.1	2.91	4.60	2.63	203.0	Not Done	96.00	3.0	
45	Female	24		OSAS	9	36	12	25	15	126	9.2	4.33	4.52	2.13	86.9	Not Done	91.00	1.2	
63	Male	29		Left Sided Heart Dx; OSAS; COPD	9	34	18	25	20	135	6.8	2.90	7.76	3.33	136.9	Not Done	94.00	2.2	
71	Male	6		Diastolic Dysfunction	7	33	11	23	12	156	6	2.88	5.13	2.46	146.0	Not Done	98.00	2.2	
70	Male	4		COPD	6	12	10	22	7	170	5.1	2.78	4.14	2.26	235.0	Not Done	97.00	al	
74	Female	9		PAH	1	33	15	22	10	134	6.7	3.60	5.18	2.79	143.0	Not Done	95.00	1.0	
72	Male	26		COPD	3	35	14	22	6	113	6.1	2.76	7.23	3.27	209.0	Not Done	91.00	1.1	
74	Male	11		CAD	4	37	5	21	11	145	6.4	2.91	6.25	2.84	125.0	Not Done	96.00	2.0	
58	Male	14		Right Heart Failure	16	33	14	20	15	100	4.1	1.99	4.72	2.29	97.5	Not Done	94.00	N/A	exclude sample
56	Female	20		COPD	14	15	14	20	14	146	6.5	3.10	5.58	2.66	73.8	Not Done	99.00	al	

27	Female	21	2	Lupus	8	23	18	20	18	138	5.7	2.40	5.50	2.31	28.0	Not Done	95.00	olic
73	Female	16	2	Scleroderma (CREST)	7	29	9	18	14	128	3.9	2.76	7.55	5.34	82.0	Not Done	96.00	1.2
79	Female	5	3	Polymyositis	0	29	9	17	7	154	6	3.22	9.27	4.97	86.3	Not Done	98.00	2.2
57	Female	35	2	None	1	25	8	15	5	139	6	3.47	4.44	2.56	133.0	Not Done	95.00	al
40	Male	39	2	None	6	22	10	15	9	112	5.9	3.13	4.14	2.20	81.3	Not Done	95.00	al
38	Female	8	2	Dyspnea; Syncope	4	21	8	14	5	122	7	3.85	5.15	2.83	102.0	Not Done	97.00	al

Characteristics of PH patients and the control subjects. Forty consecutive patients were undergoing right heart catheterization for diagnosis of pulmonary hypertension. The detail hemodynamic parameters are shown in the table.
